# TETyper: a bioinformatic pipeline for classifying variation and genetic contexts of transposable elements from short-read whole-genome sequencing data

**DOI:** 10.1099/mgen.0.000232

**Published:** 2018-11-22

**Authors:** Anna E. Sheppard, Nicole Stoesser, Ian German-Mesner, Kasi Vegesana, A. Sarah Walker, Derrick W. Crook, Amy J. Mathers

**Affiliations:** ^1^​ Nuffield Department of Medicine, University of Oxford, Oxford, UK; ^2^​ National Institute for Health Research Health Protection Research Unit in Healthcare Associated Infections and Antimicrobial Resistance, University of Oxford, Oxford, UK; ^3^​ Health Information & Technology, University of Virginia Health System, Charlottesville, Virginia, USA; ^4^​ Division of Infectious Disease and International Health, Department of Medicine, University of Virginia Health System, Charlottesville, Virginia, USA; ^5^​ Clinical Microbiology Laboratory, Department of Pathology, University of Virginia Health System, Charlottesville, Virginia, USA

**Keywords:** antimicrobial resistance (AMR), mobile genetic element (MGE), transposable element (TE), transposon, whole-genome sequencing (WGS), *Klebsiella pneumoniae* carbapenemase (KPC)

## Abstract

Much of the worldwide dissemination of antibiotic resistance has been driven by resistance gene associations with mobile genetic elements (MGEs), such as plasmids and transposons. Although increasing, our understanding of resistance spread remains relatively limited, as methods for tracking mobile resistance genes through multiple species, strains and plasmids are lacking. We have developed a bioinformatic pipeline for tracking variation within, and mobility of, specific transposable elements (TEs), such as transposons carrying antibiotic-resistance genes. TETyper takes short-read whole-genome sequencing data as input and identifies single-nucleotide mutations and deletions within the TE of interest, to enable tracking of specific sequence variants, as well as the surrounding genetic context(s), to enable identification of transposition events. A major advantage of TETyper over previous methods is that it does not require a genome reference. To investigate global dissemination of *Klebsiella pneumoniae* carbapenemase (KPC) and its associated transposon Tn*4401*, we applied TETyper to a collection of over 3000 publicly available Illumina datasets containing *bla*_KPC_. This revealed surprising diversity, with over 200 distinct flanking genetic contexts for Tn*4401*, indicating high levels of transposition. Integration of sample metadata revealed insights into associations between geographic locations, host species, Tn*4401* sequence variants and flanking genetic contexts. To demonstrate the ability of TETyper to cope with high-copy-number TEs and to track specific short-term evolutionary changes, we also applied it to the insertion sequence IS*26* within a defined *K. pneumoniae* outbreak. TETyper is implemented in python and is freely available at https://github.com/aesheppard/TETyper.

## Data Summary

1. TETyper source code has been deposited in GitHub (url – https://github.com/aesheppard/TETyper).

**Impact Statement**

Whole-genome sequencing (WGS) of bacterial pathogens has revolutionised the analysis of global and within-outbreak transmission pathways. However, the study of antibiotic resistance dissemination is more challenging, as resistance genes are often associated with mobile genetic elements (MGEs) that enable gene exchange between different host bacteria. Therefore, standard WGS approaches that focus on host strain relationships may not be informative for understanding resistance gene dissemination. We have developed a bioinformatic tool for analysing WGS data from the perspective of a specific MGE–resistance gene association. The outputs produced identify variation within the MGE, as well as signatures of MGE mobility. This information can then be used to track the movement of the resistance gene, thus overcoming previous limitations by defining relationships from a resistance gene perspective, rather than a host-strain perspective. In an epidemiological context, this can provide insight into specific transmission pathways, thus informing infection control within outbreak scenarios, as well as increasing our understanding of global pathways of resistance dissemination.

## Introduction

Increasing antibiotic resistance in a range of bacterial pathogens is a major global health threat, but our understanding of resistance gene dissemination remains incomplete. Many resistance genes are carried on mobile genetic elements (MGEs), enabling bacteria to evolve in response to antimicrobial pressures via gene exchange. MGEs of relevance include plasmids, which are extrachromosomal, usually circular, DNA structures that can be transferred between different host bacteria, and transposable elements (TEs), which are short stretches of DNA, often carried on plasmids, that can autonomously mobilise to new genomic locations via transposition [1–3]. TEs comprise transposons, which carry additional cargo genes, such as antibiotic-resistance genes, and insertion sequences (ISs), which comprise only elements necessary for transposition; however, composite structures involving ISs can also be involved in resistance gene mobilisation [4, 5].

Whole-genome sequencing (WGS) has revolutionised the analysis of pathogen transmission by enabling high-resolution insight into chromosomal relatedness [6–9]. However, resistance gene dissemination via MGEs is more complicated because horizontal transfer disrupts pairing between resistance genes and host strains. To assess relatedness from the perspective of a mobile resistance gene, it is necessary to examine the gene’s genetic context. However, the most widely used WGS technologies (e.g. Illumina) produce short sequencing reads; these result in fragmented assemblies, with resistance genes often present on very short contigs due to associations with repetitive elements such as TEs. This makes tracking the associated plasmids largely impractical, as assembling complete plasmid sequences from short reads is problematic [10], and reference-based approaches can be unreliable due to transposition or homologous recombination disrupting pairing between host plasmids and resistance genes [11]. Alternatively, if a resistance gene has a stable association with a specific transposon, then tracking the transposon may be a better proxy for understanding resistance gene dissemination.

One example of such an association is the *Klebsiella pneumoniae* carbapenemase (KPC) gene *bla*_KPC_ and its associated replicative transposon Tn*4401. bla*_KPC_ was first identified in the USA in 1996, and has since spread globally, being responsible for a large proportion of carbapenem-resistant Enterobacteriaceae infections worldwide [12–15]. Initially, it was largely associated with *K. pneumoniae* multi-locus sequence type (ST) 258 and the IncFII plasmid pKpQIL, but it has since spread to various other plasmids, other *K. pneumoniae* strains, other species of Enterobacteriaceae, and occasionally non-Enterobacteriaceae [11, 16–18]. Given the importance of Tn*4401* transposition in facilitating this spread, the ability to track transposition events and sequence variation within Tn*4401* may be helpful for better understanding *bla*_KPC_ dissemination.

A similar tracking approach may also be useful for investigating the evolution of other TEs of interest. For example, replicative intermolecular transposition (where the target site for transposition is from a different DNA molecule) of a variety of TEs, including Tn*4401*, involves target site duplication (TSD). This results in short (~2–14 bp) direct repeats flanking the newly transposed copy [3]. Intramolecular transposition (where the transposition target site is part of the same DNA molecule) disrupts these flanking repeats, and investigation of TSD sequences can be used to gain insight into historical transposition events, as has been demonstrated for the widely dispersed IS*26* [19].

Most available tools for characterising TE mobilisation from WGS data rely on detecting variation relative to a reference sequence (e.g. [20–23]). While this may be appropriate for eukaryotic (and some prokaryotic) genomes, bacterial TEs are often carried on plasmids for which no suitable references are available. ISMapper [20] was developed specifically for detecting TE insertion sites in bacterial genomes, and can be run in two modes, using either a reference genome as input, or a *de novo* assembly of the sample of interest. In the latter mode, it predicts which contigs are linked to the TE of interest, but does not provide detailed information on sequence variation within the TE or base-pair resolution of insertion sites. To provide a tool for characterising TE insertion sites and sequence variation from short-read WGS data, without relying on a genome reference, and producing easily comparable outputs for epidemiological tracking purposes, we developed TETyper. TETyper takes raw sequencing reads as input, and identifies: 1. Structural variation within a specific TE of interest, 2. Single-nucleotide variants (SNVs) within the TE, and 3. Flanking genetic context(s) of the TE. Variation within the TE captures signatures of micro-evolution, while the flanking sequences capture signatures of transposition, as every transposition event introduces a new genetic sequence context for the TE. This information can then be utilised for investigating transposition pathways, as well as gaining epidemiological insight in the context of resistance gene dissemination, both within outbreaks and globally. We demonstrate the utility of TETyper by applying it to a collection of over 3000 publicly available Illumina datasets containing *bla*_KPC_, and to IS*26* within a clonal *K. pneumoniae* outbreak.

## Theory and implementation

### Description of the TETyper pipeline

**Fig. 1.**
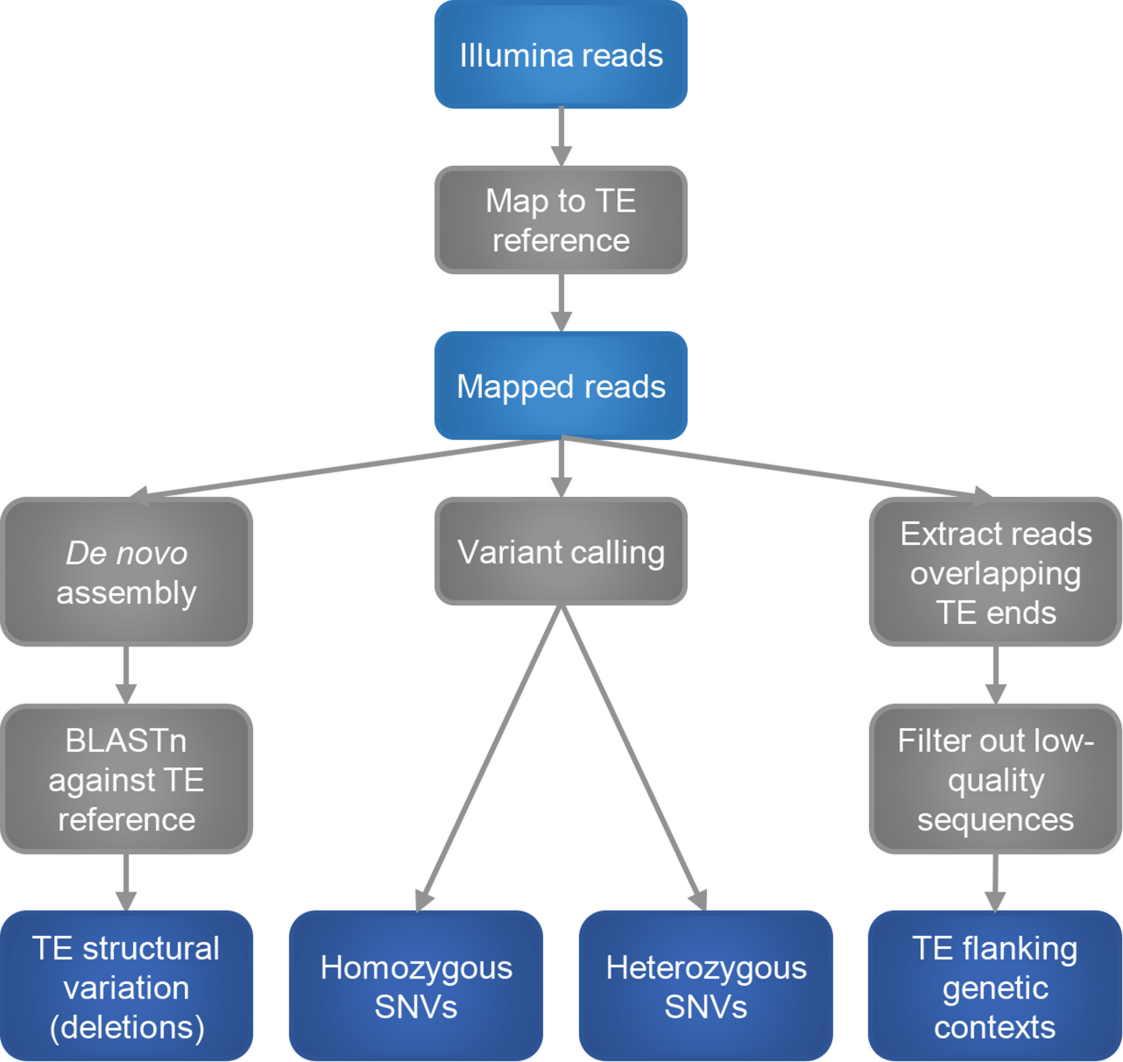
Overview of processing steps in the TETyper pipeline.

An overview of processing steps is shown in Fig. 1. Firstly, reads are mapped against a reference representing the TE of interest using bwa mem with default parameters [24]. For the remaining steps, only mapped reads are retained.

To classify structural variation within the TE, we focus on deletions relative to the reference, since insertions and other rearrangements are difficult to classify reliably using short-read data. This is achieved by assembling the reads that map to the TE (along with any unmapped pairs) using spades [25], followed by BLASTn [26] to identify missing regions.

To identify SNVs, variant calling is performed under a diploid model using samtools mpileup [27], with variants excluded if they fall within a deleted region as identified above, or if they are not supported by at least one read in each direction. Heterozygous variants are assumed to represent within-sample mixtures (i.e. multiple, slightly different, copies of the TE), and are reported along with the number of reads supporting each nucleotide.

To identify flanking genetic context(s) of the TE, the user specifies the desired length of flanking sequence to classify, which should be short enough that sequencing errors are rare. Reads mapping to the start/end of the TE sequence are examined, and the sequence of each read immediately adjacent to the start/end of the TE reference, of the length specified above, is extracted. To remove low-quality sequences, reads are discarded if the mapped sequence is too short (default threshold 30 bp), or if the minimum base quality within the flanking sequence is too low (default threshold 10). The resulting flanking sequences are then output if they pass additional thresholds for the number of supporting reads (defaults: 10 total, 1 each strand). In this way, if there is a single copy of the TE present in the sample, there should be a single flanking sequence identified at each end of the TE. On the other hand, every time a TE undergoes transposition, it inserts into a new genetic context. Therefore, if there are X copies of a TE in a sample, then there will be up to X distinct flanking sequences at each end of the TE. For the applications below, we examine flanking sequences equal to TSD length (5 bp for Tn*4401* and 8 bp for IS*26*), thus providing a simple signature for identifying shared insertion sites. For other applications, if the wider genetic context is of interest, then longer flanks can be used to obtain unique sequences for comparison with a reference genome.

Specific parameters used for running TETyper are described in Supplementary Methods.

### Validation

The TETyper output was validated using a subset of isolates from the datasets described below for which complete, closed references were previously generated using long-read sequence data [11, 28–30].

For the *bla*_KPC_ dataset, we used 24 isolates representing a range of species, with different Tn*4401* variants, and 1–2 Tn*4401* copies each [11, 28, 29]. In all cases, the TETyper output was consistent with the Tn*4401* elements present in the long-read assemblies (Table S2, available in the online version of this article).

Interestingly, for three of the six isolates with two Tn*4401* copies (CAV1217, CAV1321 and CAV1741), the immediate Tn*4401* flanking sequence was identical between the two copies, presumably because duplication of Tn*4401* occurred via a mechanism other than Tn*4401* transposition that also involved duplication of the surrounding sequence (e.g. via an additional flanking TE, as previously described for CAV1217 [28]). In these cases, TETyper only identified a single left/right flanking sequence and if this output were taken at face value, then the number of Tn*4401* copies would be underestimated. This highlights the way in which TETyper's characterisation of the immediate flanking sequences is specifically designed to capture transposition of the reference TE, while other types of genetic mobility may be missed. In order to capture mobilisation events involving an additional flanking TE, TETyper could be used with the entire nested TE structure as a reference; however, this would require knowledge of the relevant TE structures involved.

For the other three isolates with two Tn*4401* copies (CAV1042, CAV1392 and CAV1596), the immediate flanking sequences were different, and TETyper correctly identified two flanking sequences in each case. For one of these isolates (CAV1392), the coverage of the flanking sequences was sufficiently different that this information could be used to phase the left and right flanking sequences in the absence of long-read data.

For the IS*26* analysis, there was one isolate that had previously been long-read sequenced (PMK1; accession CP008929.1-CP008933.1). There were six full-length and three truncated copies of IS*26* in the long-read assembly, resulting in seven and eight left and right flanking genetic contexts respectively (Table S3). The flanking sequences identified by TETyper (Fig. 4) were a perfect match, demonstrating the ability of TETyper to accurately identify flanking genetic contexts even in the presence of multiple TE copies.

It is worth noting that the TETyper output did not identify any deletions, even though truncated IS*26* copies were present. This is expected, as when the multiple copies of IS*26* are combined, there are no missing regions. Nevertheless, it is important to be aware that the method may be unable to capture structural variation when multiple copies are present.

On the other hand, one of the IS*26* copies in the long-read assembly had a single SNV relative to the IS*26* reference at position 107, resulting in a missense mutation in the IS*26* transposase. However, TETyper did not identify any SNVs. This is a genuine discrepancy, as the heterozygous SNV output is designed to capture situations where multiple copies of the TE have SNV-level differences. However, in this case only one out of eight IS*26* copies that cover the variant position has the mutation, and consistent with this the read pileup generated from mapping Illumina reads to the IS*26* reference had only 12 % of reads at this position with the variant base. This highlights a limitation in the variant-calling algorithm used, as alternative alleles present at low frequency may not be detectable. Interestingly, all 34 samples showed similar frequencies of the variant base at this position, consistent with the variant allele being present across all samples, as indicated by the presence of the corresponding flanking sequences (ATTGTTTT/GGTCTTAA) in all samples (Fig. 4). For two samples (PMK21b and PMK25), the site was in fact called as heterozygous, indicating that the composition of IS*26* elements within this sample collection sits close to the limit of detection for identifying heterozygous SNVs (as defined by the samtools variant-calling algorithm).

### Application to Tn*4401*

To demonstrate the utility of TETyper for epidemiological investigations, we applied it to 3054 *bla*_KPC_-positive Illumina datasets retrieved from a December 2016 snapshot of the European Nucleotide Archive [31] (Supplementary Methods, Table S1). The Tn*4401*b sequence from pKPC_UVA01 (CP017937) was used as a reference.

#### Structural variation in Tn*4401*

**Fig. 2.**
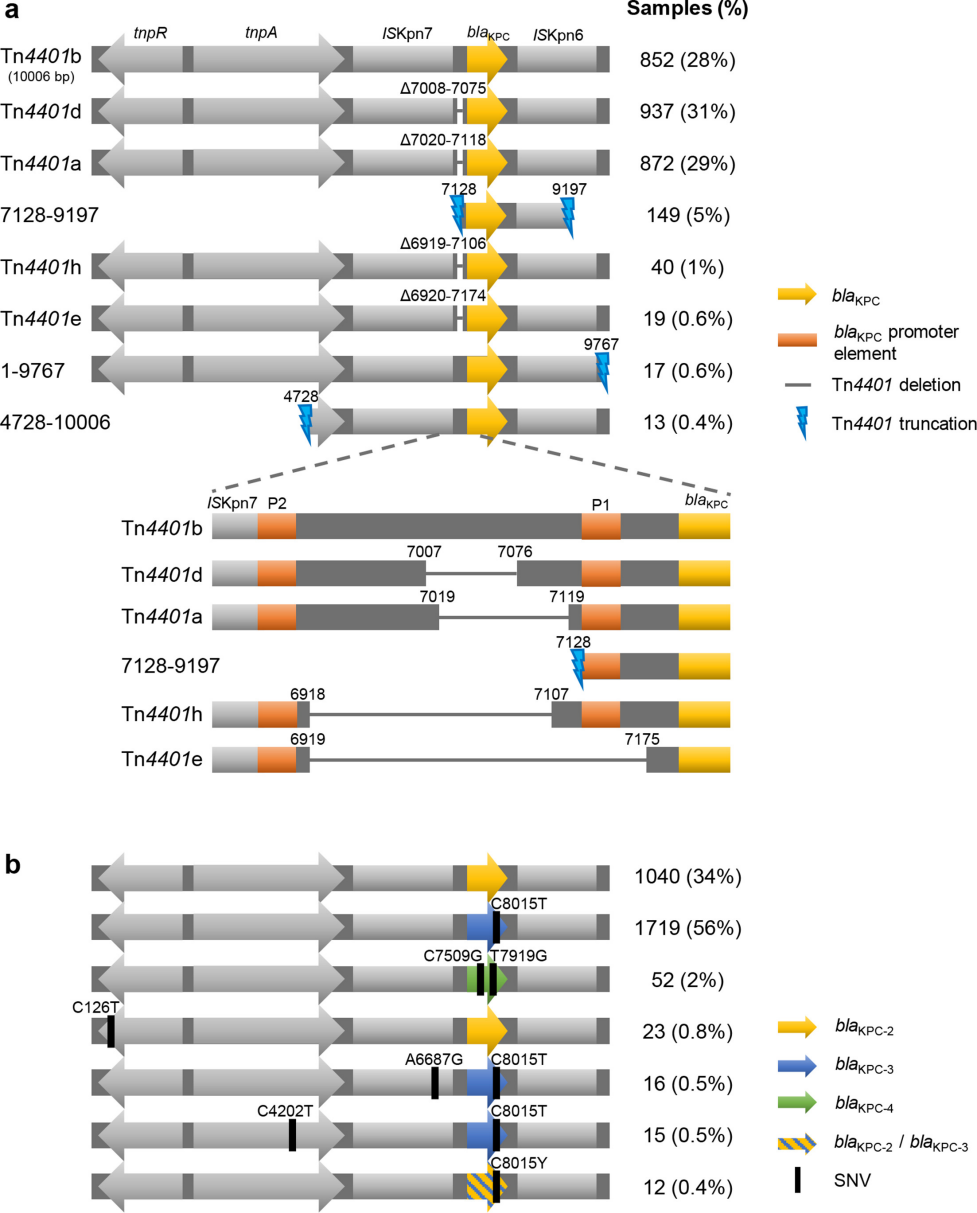
Structure of Tn*4401* showing common structural variants (a) and SNVs (b). Only variants found in at least ten samples are shown. For simplicity, SNV variants are all illustrated within a Tn*4401*b structural background.

There were eight ‘common’ (found in ≥10 samples) structural variants of Tn*4401* (Fig. 2a). The ancestral Tn*4401*b structure [32] was found in 852 out of 3054 (28 %) samples. Four variants represented different deletions immediately upstream of *bla*_KPC_, namely Tn*4401*d [33, 34], Tn*4401*a [32], Tn*4401*h [35] and Tn*4401*e [33, 34] in 937 (31 %), 872 (28 %), 40 (1.3 %) and 19 (0.6 %) samples respectively. The other three variants all represented truncations of Tn*4401*.

Deletions affecting the promoter region upstream of *bla*_KPC_ have been shown to result in increased expression [34, 35], which is expected to be advantageous under antibiotic selection pressure. As several of these were observed, with no other common internal deletions across the 10 kb Tn*4401* sequence, this indicates that much of the structural variation observed may be due to selection rather than random genetic drift. Truncation of Tn*4401* presumably prevents further transposition; one possible reason for the abundance of truncation variants is that they bring other TEs into the vicinity of *bla*_KPC_ [36], thus providing alternative routes for gene mobilisation.

**Fig. 3.**
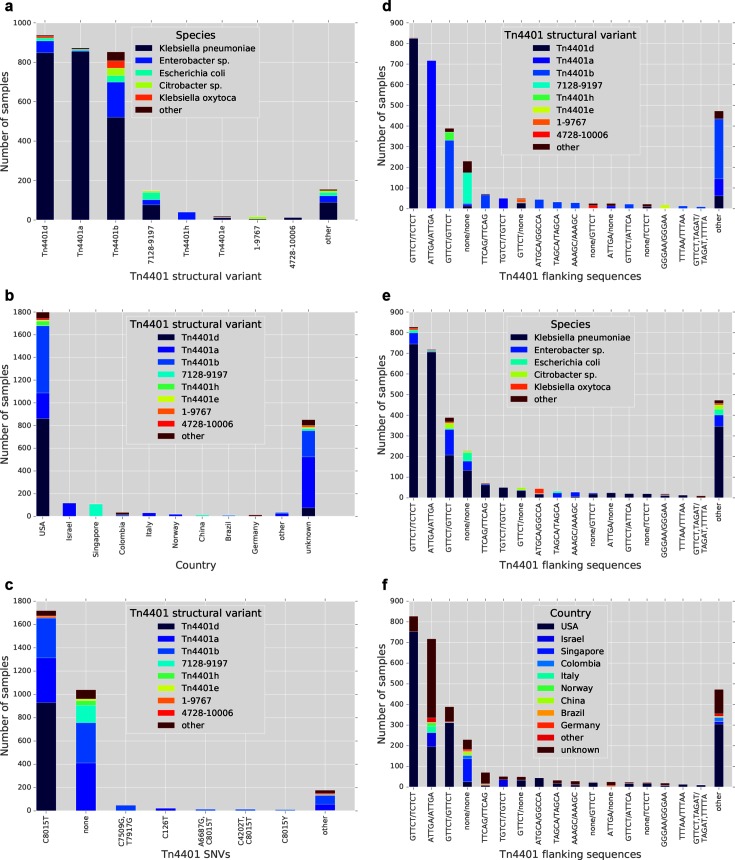
Associations between Tn*4401* structural variants, SNVs, flanking genetic contexts, host species, and countries of origin amongst a collection of 3054 *bla*_KPC_ samples from the European Nucleotide Archive. (a) Distributions of host species for different structural variants of Tn*4401*. (b) Distributions of Tn*4401* structural variants for different countries of origin. (c) Distributions of Tn*4401* structural variants for different Tn*4401* SNVs. (d–f) Distributions of Tn*4401* structural variants (d), host species (e) and countries of origin (f) for different Tn*4401* flanking genetic contexts.

Specific structural variants were generally found in multiple host species, indicating wide horizontal dissemination via inter-species transfer (Figs 3a and S1a). Tn*4401*b was the most widely disseminated, being found in ten different genera, while Tn*4401*a was relatively restricted to *K. pneumoniae* (98 %). Several different structural variants were present in samples from the USA, while samples from other countries generally had a single predominant variant (Figs 3b and S1b), supporting the origination and diversification of *bla*_KPC_ and Tn*4401* in Enterobacteriaeceae in the USA. However, the dataset was heavily biased towards isolates from the USA, and for 851 out of 3054 (28 %) samples the country of origin was unknown, highlighting limitations in metadata availability.

#### Single-nucleotide variation in Tn*4401*

Most SNV variation involved sites within the *bla*_KPC_ gene (Fig. 2b), again implicating selection for KPC function in explaining observed variation. The two most common variants carried *bla*_KPC-2_ and *bla*_KPC-3_, in 1040 out of 3054 (34 %) and 1719 out of 3054 (56 %) samples respectively, with each found in several different structural backgrounds (Figs 3c and S1c).

Interestingly, 12 samples showed polymorphism at the site that differentiates *bla*_KPC-2_ and *bla*_KPC-3_ (Fig. 2b), signifying a mixture of the two alleles and indicating that these samples most likely contain two copies of Tn*4401*, one with *bla*_KPC-2_ and one with *bla*_KPC-3_. Minor allele percentages ranged from 10–49 %. These occurred in several Tn*4401* structures, including Tn*4401*a, Tn*4401*b and Tn*4401*d, and several host species, including *Escherichia coli*, *K. pneumoniae*, *Klebsiella oxytoca* and *Enterobacter cloacae*, with varying flanking sequences. This indicates that the presence of multiple *bla*_KPC_ variants may be a general phenomenon, indicating repeated multiple acquisition of *bla*_KPC_ and/or repeated mutation converting *bla*_KPC-2_ to *bla*_KPC-3_ (or vice versa).

### Flanking genetic contexts of Tn*4401*

The most common 5 bp sequences flanking Tn*4401* were GTTCT/TCTCT, ATTGA/ATTGA and GTTCT/GTTCT, present in 827 (27 %), 718 (24 %) and 389 (13 %) out of 3054 samples respectively (Figs 3d–f and S1d–f). ATTGA/ATTGA corresponds to the epidemic IncFII pKpQIL plasmid; these samples were almost exclusively Tn*4401*a-containing *K. pneumoniae*, but from a variety of geographic locations. GTTCT/GTTCT corresponds to Tn*1*/*2*/*3*-like elements (including Tn*1331*; see below), which have been described as containing Tn*4401* in many different plasmid backbones [11]; these samples represented a wider variety of Tn*4401* structures and host species. GTTCT/TCTCT is consistent with IncFIA pBK30661/pBK30683-like plasmids, where Tn*4401* is adjacent to a partial Tn*1331* element on the left side only, presumably as a result of deletion on the right side following initial integration [37].

For 398 out of 3054 (13 %) samples, there was no flanking sequence identified on one or both sides of Tn*4401*, consistent with Tn*4401* truncation. These truncated Tn*4401* structures are presumably not transpositionally active, so TETyper would be unable to capture ongoing mobilisation events in these cases. For the vast majority, however, Tn*4401* appeared to be intact; 2368 (78 %) samples had a single flanking sequence identified on each side of Tn*4401*, indicating a single intact copy, and 288 (9 %) had multiple flanking sequences on one or both sides, indicating multiple copies.

Of those with a single copy, 1417 out of 2368 (60 %) had the same 5 bp sequence at both ends of Tn*4401*, consistent with TSD following standard intermolecular transposition. Surprisingly, 951 out of 2368 (40 %) showed different 5 bp sequences, indicating disruption of TSD signatures and suggesting that intramolecular transposition of Tn*4401* or other rearrangements disrupting TSDs may be relatively common.

Altogether, there were 193 and 213 distinct 5 bp sequences flanking the left and right sides of Tn*4401* respectively, and a total of 272 distinct flanking sequence profiles, indicating relatively frequent transposition. This diversity indicates that the classification of flanking genetic contexts in this way may be useful for epidemiological tracking by providing higher genetic resolution than strain typing alone.

### Application to IS*26*

**Fig. 4.**
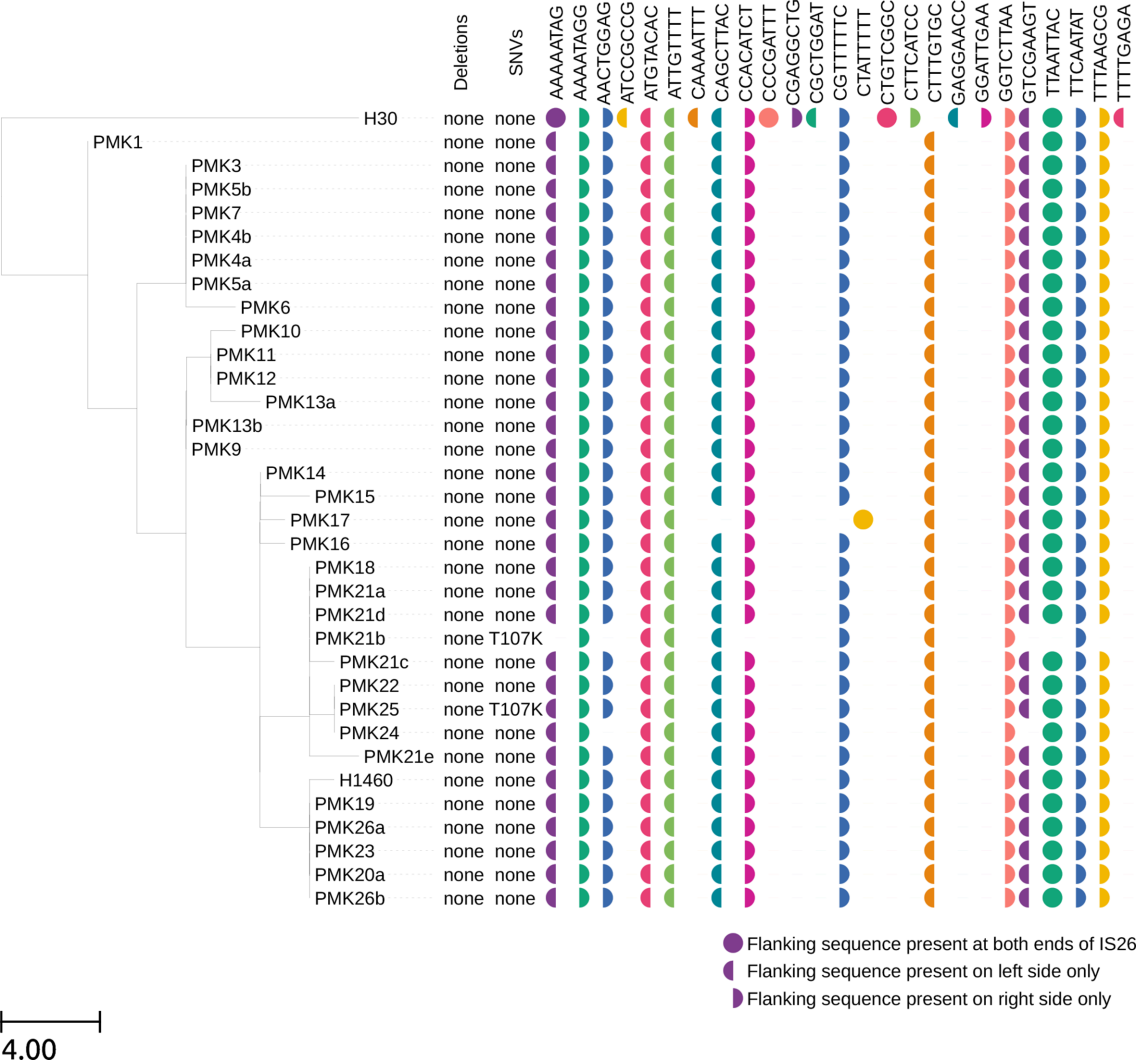
Variation in IS*26* amongst 34 ST15 *K. pneumoniae* isolates from an NDM-1 outbreak. TETyper output is annotated alongside a maximum likelihood phylogeny that was generated using IQ-TREE version 1.3.13 [38], after mapping to the MGH78578 reference as previously described [30]. Branch lengths are shown as SNVs per genome.

To demonstrate the utility of TETyper for analysing specific TE mobility events without relying on a reference genome, we applied it to IS*26* for 34 closely related *K. pneumoniae* ST15 isolates from an NDM-1 outbreak in Nepal [30]. These isolates varied in the number and sequence of genetic contexts of IS*26*, with evidence for 4–14 copies per isolate (Fig. 4, Table S1). In some cases, the TETyper output provided higher genetic resolution than a standard phylogenetic approach, with IS*26* flanking sequence profiles differing between pairs of isolates with 0 chromosomal SNVs (Fig. 4; PMK21b vs PMK18/21a/21d and PMK24 vs PMK22/25).

### Conclusion

We have developed a novel bioinformatic pipeline, TETyper, for classifying sequence variation and flanking genetic contexts of TEs from short-read WGS data, without requiring a genome reference. We have demonstrated the utility of TETyper by applying it to Tn*4401* for a large, global *bla*_KPC_ collection, as well as IS*26* within a small, defined outbreak. This revealed surprising diversity in both cases, and provided insights into patterns of transposition and mutational change within Tn*4401*. In an epidemiological context, the within-TE variation and transposition signatures identified by TETyper could be used to facilitate higher-resolution resistance gene tracking related to gene mobility than is currently possible using other WGS-based methods.

## Data bibliography

1. TETyper source code, Sheppard AE, GitHub, (2018).

## Supplementary Data

Supplementary File 1

Supplementary File 2
