# Supplementary material for: TETyper: a bioinformatic pipeline for classifying variation and genetic contexts of transposable elements from short-read whole-genome sequencing data
Source: Microb Genom. 2018 Nov 22;4(12):e000232. doi: 10.1099/mgen.0.000232 (PMC6412039; doi:10.1099/mgen.0.000232)
Supplement: Supplementary File 2 [file mgen-4-232-s001.pdf]

Supplementary Methods: Description of *bla*$_{KPC}$ sample collection and parameters for running TETyper

To identify short-read datasets containing *bla*$_{KPC}$, we queried a snapshot of all bacterial WGS datasets from the European Nucleotide Archive from December 2016, using http://bigsi.io (1), with *bla*$_{KPC-2}$ as a query sequence and a query kmer threshold of 40%. This returned 3120 results.

Metadata for these sequencing datasets was retrieved using Biopython's Entrez package. Based on this, we excluded non-Illumina datasets (n=48; 37 454, 10 PacBio, 1 Ion Torrent) and single-read datasets (n=3). A further three datasets were excluded following sequence download (only had single-read data available, n=2; unavailable for download, n=1), leaving 3066 datasets for comparison.

These 3066 paired-end Illumina datasets were run through the TETyper pipeline, with parameters according to the following example command (Tn4401b-1.fasta, struct_profiles.txt and snp_profiles.txt are provided in the github repository at https://github.com/aesheppard/TETyper):
TETyper.py --fq1 ERR025634_1.fastq.gz --fq2 ERR025634_2.fastq.gz --outprefix ERR025634 --ref Tn4401b-1.fasta --flank_len 5 --struct_profiles struct_profiles.txt --snp_profiles snp_profiles.txt --verbosity 2 --show_region 7202-8083

12 of these 3066 datasets were classified as *bla*$_{KPC}$-negative, as there was either no full length *bla*$_{KPC}$ gene present in the *de novo* assembly produced (n=4), or the *de novo* assembly step failed due to a low number of reads mapping to the Tn*4401* reference (n=8). These were excluded from further processing, leaving a total of 3054 *bla*$_{KPC}$-positive sequencing datasets for analysis.

For the IS*26* analysis, samples were run according to the following example command (IS26.fasta is provided in the github repository and represents a trimmed version of GenBank accession X00011.1):
TETyper.py --fq1 PMK1_1.fq.gz --fq2 PMK1_2.fq.gz --outprefix PMK1 --ref IS26.fasta --verbosity 2 --flank_len 8

References

1. Bradley P, den Bakker H, Rocha E, McVean G, Iqbal Z. Real-time search of all bacterial and viral genomic data. bioRxiv. 2017.

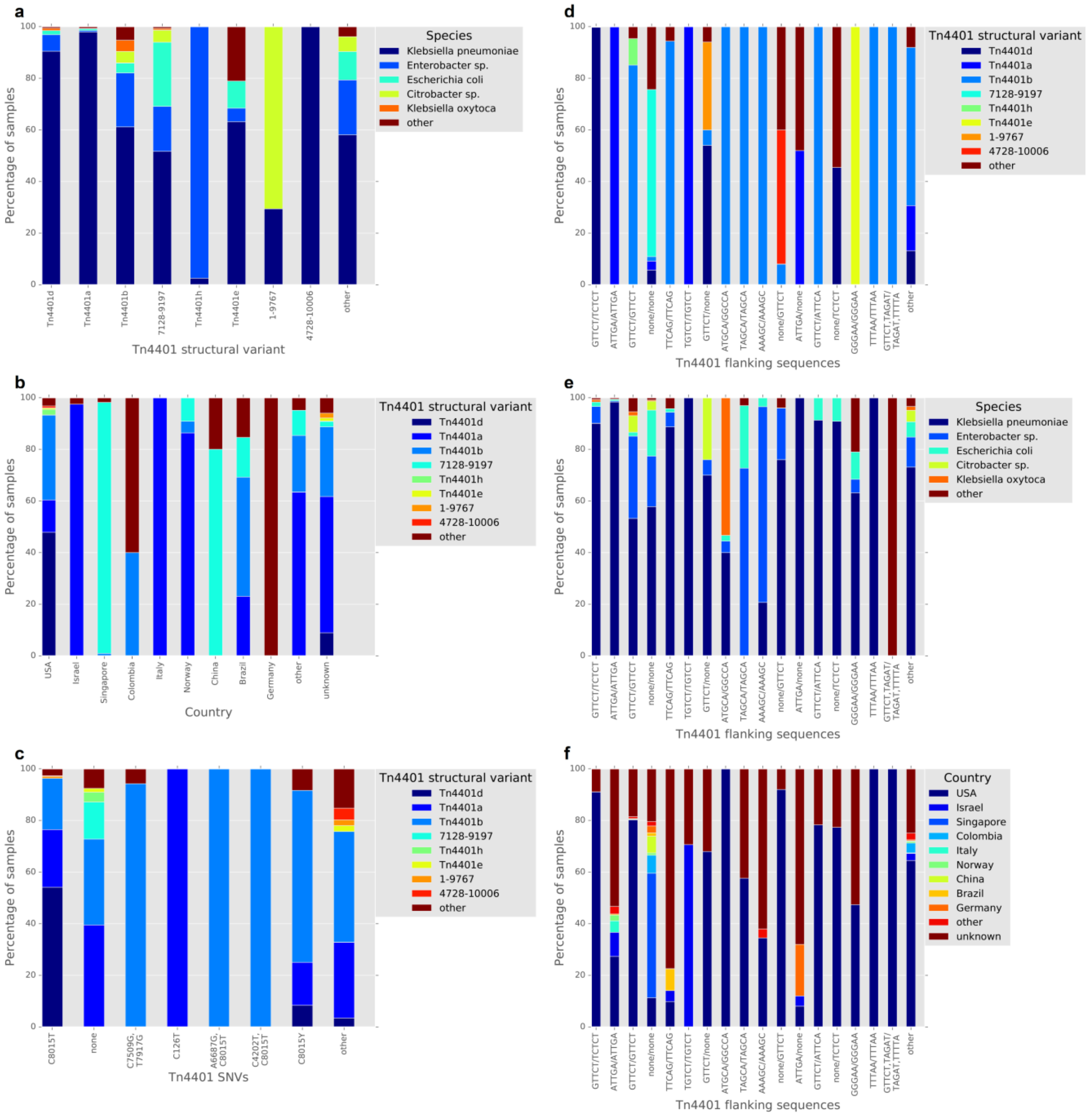

Fig. S1: Associations between Tn*4401* structural variants, SNVs, flanking genetic contexts, host species, and countries of origin amongst a collection of 3054 *bla*$_{\text{KPC}}$ samples from the European Nucleotide Archive, shown as proportions. (a) Distributions of host species for different structural variants of Tn*4401*. (b) Distributions of Tn*4401* structural variants for different countries of origin. (c) Distributions of Tn*4401* structural variants for different Tn*4401* SNVs. (d-f) Distributions of Tn*4401* structural variants (d), host species (e) and countries of origin (f) for different Tn*4401* flanking genetic contexts.

Table S2. Validation of TETyper output for 24 *bla*$_{KPC}$ isolates with complete long-read assemblies available.

Isolate	Species	Assembly accession	Tn*4401* elements in long-read assembly	SRA accession	Tn*4401* Structure	Tn*4401* SNVs	Left flanks (coverage)	Right flanks (coverage)	Agreement
CAV1016	Klebsiella pneumoniae	CP017934.1-CP017937.1	GTTCT-Tn4401b-GTTCT	SRR1582858	Tn4401b	none	GTTCT (129)	GTTCT (95)	Yes
CAV1042	Klebsiella pneumoniae	CP018665.1-CP018671.1	ATATC-Tn4401b-AATAT, GTTCT-Tn4401b-GTTCT	SRR1582861	Tn4401b	none	ATATC (73), GTTCT (91)	AATAT (69), GTTCT (61)	Yes
CAV1043	Enterobacter asburiae	CP011585.1-CP011591.1	GTTCT-Tn4401b(C8015T)-GTTCT	SRR2965752	Tn4401b	C8015T	GTTCT (108)	GTTCT (81)	Yes
CAV1099	Klebsiella oxytoca	CP011592.1-CP011597.1	ATGCA-Tn4401b-GGCCA	SRR2965639	Tn4401b	none	ATGCA (107)	GGCCA (79)	Yes
CAV1151	Kluyvera intermedia	CP011598.1-CP011602.1	GTTCT-Tn4401b-GTTCT	SRR2965721	Tn4401b	none	GTTCT (102)	GTTCT (63)	Yes
CAV1176	Enterobacter hormaechei	CP011658.1-CP011662.1	GTTCT-Tn4401h-GTTCT	SRR2965806	Tn4401h	none	GTTCT (104)	GTTCT (76)	Yes
CAV1193	Klebsiella pneumoniae	CP013321.1-CP013326.1	GTTCT-Tn4401b-GTTCT	SRR2965672	Tn4401b	none	GTTCT (85)	GTTCT (86)	Yes
CAV1217	Klebsiella pneumoniae	CP018672.1-CP018676.1	ATGAA-Tn4401b(C7509G,T7917G)-ATGAA, ATGAA-Tn4401b(C7509G,T7917G)-ATGAA	SRR1582870	Tn4401b	C7509G, T7917G	ATGAA (56)	ATGAA (55)	Yes
CAV1311	Enterobacter cloacae	CP011569.1-CP011572.1	GTTCT-Tn4401h-GTTCT	SRR2965815	Tn4401h	none	GTTCT (94)	GTTCT (92)	Yes
CAV1320	Klebsiella aerogenes	CP011573.1-CP011574.1	TTGTT-Tn4401b-TTGTT	SRR2965748	Tn4401b	none	TTGTT (209)	TTGTT (168)	Yes
CAV1321	Citrobacter freundii	CP011603.1-CP011612.1	GTTCT-Tn4401b-GTTCT, GTTCT-Tn4401b-GTTCT	SRR2965690	Tn4401b	none	GTTCT (150)	GTTCT (133)	Yes
CAV1335	Klebsiella oxytoca	CP011613.1-CP011618.1	ATGCA-Tn4401b-GGCCA	SRR2965660	Tn4401b	none	ATGCA (57)	GGCCA (30)	Yes
CAV1344	Klebsiella pneumoniae	CP011619.1-CP011624.1	GTTCT-Tn4401b-GTTCT	SRR1582875	Tn4401b	none	GTTCT (37)	GTTCT (29)	Yes
CAV1374	Klebsiella oxytoca	CP011625.1-CP011636.1	GTTCT-Tn4401b-GTTCT	SRR2965655	Tn4401b	none	GTTCT (117)	GTTCT (88)	Yes
CAV1392	Klebsiella pneumoniae	CP011575.1-CP011578.1	AGATA-Tn4401b(C8015T)-AGATA, GTTCT-Tn4401b(C8015T)-GTTCT	SRR1582895	Tn4401b	C8015T	AGATA (56), GTTCT (151)	AGATA (52), GTTCT (101)	Yes
CAV1411	Enterobacter cloacae	CP011579.1-CP011581.1	GTTCT-Tn4401h-GTTCT	SRR2965820	Tn4401h	none	GTTCT (87)	GTTCT (58)	Yes
CAV1417	Klebsiella pneumoniae	CP018348.1-CP018352.1	ATGAA-Tn4401b(T6800C,C7509G,T7917G)-ATGAA	SRR2965682	Tn4401b	T6800C, C7509G, T7919G	ATGAA (73)	ATGAA (53)	Yes
CAV1453	Klebsiella pneumoniae	CP018353.1-CP018356.1	AATAA-Tn4401a-CTATT	SRR2965692	Tn4401a	none	AATAA(101)	CTATT (30)	Yes
CAV1492	Serratia marcescens	CP011637.1-CP011642.1	TTTTT-Tn4401b(T9663C)-TTTTT	SRR2965730	Tn4401b	T9663C	TTTTT (112)	TTTTT (76)	Yes
CAV1596	Klebsiella pneumoniae	CP011643.1-CP011647.1	GTTCT-Tn4401b(C8015T)-GTTCT, TATCG-Tn4401b(C8015T)-TATCG	SRR1582868	Tn4401b	C8015T	GTTCT (157), TATCG (200)	GTTCT (110), TATCG (157)	Yes
CAV1668	Enterobacter cloacae	CP011582.1-CP011584.1	GTTCT-Tn4401h-GTTCT	SRR2965612	Tn4401h	none	GTTCT (144)	GTTCT (90)	Yes
CAV1669	Enterobacter cloacae	CP011648.1-CP011650.1	GTTCT-Tn4401h-GTTCT	SRR2965616	Tn4401h	none	GTTCT (103)	GTTCT (84)	Yes
CAV1741	Citrobacter freundii	CP011651.1-CP011657.1	GTTCT-Tn4401b-GTTCT, GTTCT-Tn4401b-GTTCT	SRR2965739	Tn4401b	none	GTTCT (256)	GTTCT (191)	Yes
CAV1752	Klebsiella oxytoca	CP018357.1-CP018362.1	AACAA-Tn4401b-AACAA	SRR2965667	Tn4401b	none	AACAA (74)	AACAA (45)	Yes

Table S3. IS*26* elements present in PMK1 long-read assembly.

Contig	Start position	End position	Length	Deletions	SNVs	Left flank	Right flank	Flanks correctly identified by TETyper
CP008930.1	36476	37211	736	1-84	none		CGTTTTTC	Yes
CP008930.1	40738	41347	610	611-820	none	CAGCTTAC		Yes
CP008930.1	45939	45120	820	none	none	CTTTGTGC	AAAATAGG	Yes
CP008930.1	62346	63165	820	none	T107G	ATTGTTTT	GGTCTTAA	Yes
CP008933.1	38164	38983	820	none	none	AAAAATAG	CCACATCT	Yes
CP008933.1	41989	41170	820	none	none	GTCGAAGT	TTTAAGCG	Yes
CP008933.1	43685	42866	820	none	none	TTAATTAC	AACTGGAG	Yes
CP008933.1	76011	75318	694	1-126	none		TTAATTAC	Yes
CP008933.1	205677	206496	820	none	none	ATGTACAC	TTCAATAT	Yes
